# Impact assessment of an automated drug-dispensing system in a tertiary hospital

**DOI:** 10.6061/clinics/2017(10)07

**Published:** 2017-10

**Authors:** Débora de-Carvalho, José Luiz Alvim-Borges, Cristiana Maria Toscano

**Affiliations:** I Farmacia, Hospital Sirio-Libanes (HSL), Sao Paulo, SP, BR; II Instituto de Ensino e Pesquisa, Hospital Sirio-Libanes (HSL), Sao Paulo, SP, BR; III Departamento de Saude Coletiva, Instituto de Patologia Tropical e Saude Publica, Universidade Federal de Goias, Goiania, GO, BR

**Keywords:** Pharmacy, Cost and Cost Analysis, Automated Drug-Dispensing Systems, Impact

## Abstract

**OBJECTIVE:**

To evaluate the costs and patient safety of a pilot implementation of an automated dispensing cabinet in a critical care unit of a private tertiary hospital in São Paulo/Brazil.

**METHODS:**

This study considered pre- (January-August 2013) and post- (October 2013-October 2014) intervention periods. We considered the time and cost of personnel, number of adverse events, audit adjustments to patient bills, and urgent requests and returns of medications to the central pharmacy. Costs were evaluated based on a 5-year analytical horizon and are reported in Brazilian Reals (R$) and US dollars (USD).

**RESULTS:**

The observed decrease in the mean number of events reported with regard to the automated drug-dispensing system between pre- and post-implementation periods was not significant. Importantly, the numbers are small, which limits the power of the mean comparative analysis between the two periods. A reduction in work time was observed among the nurses and administrative assistants, whereas pharmacist assistants showed an increased work load that resulted in an overall 6.5 hours of work saved/day and a reduction of R$ 33,598 (USD 14,444) during the first year. The initial investment (R$ 206,065; USD 88,592) would have been paid off in 5 years considering only personnel savings. Other findings included significant reductions of audit adjustments to patient hospital bills and urgent requests and returns of medications to the central pharmacy.

**CONCLUSIONS:**

Evidence of the positive impact of this technology on personnel time and costs and on other outcomes of interest is important for decision making by health managers.

## INTRODUCTION

Healthcare-related errors are common 1. In the United States between 2000 and 2002, 37 million admissions occurred in the Medicare system, and 1.14 million patient safety-related incidents were reported. In an extrapolation to all admissions in American hospitals during this period, 575,000 preventable deaths would result from patient safety-related incidents 2.

A German study addressing three public hospitals, totaling 1,208 beds and 49,462 patients showed that the mean cost of patients (n 1,891) who suffered an adverse drug event (ADE) was €5,113-€10,059. The mean hospital stay of patients with an ADE was 2.9 days longer than that of patients without an ADE. An extrapolation for the entire country estimates that ADEs entail a total annual cost of €1.058 billion 3.

A prospective observational study found that 61% (170/277) of serious medical errors – which cause or have the potential to cause damage or injury – are drug prescription or administration errors 4. Another prospective observational study of intensive care reported one error, resulting in a potential or actual ADE, for every 5 prescription items administered 5.

The occurrence of drug prescription errors is 7%, affecting 2% of patients/day and 50% of hospital admissions 6. Conversely, a systematic review indicated that approximately 10% of errors involving drugs corresponded to administration errors 7.

In Brazil, a prospective cohort study, which was conducted in a tertiary teaching hospital with blinded nursing staff, found that 21% (238/1,119) of the doses of drugs prescribed and dispensed by the pharmacy were not administered 8.

The application of information technology to healthcare has increased the safety of hospital prescription and administration procedures. Computerized physician order-entry systems with a drug menu standardized by the hospital institution, a clinical decision support system, and electronic medication administration record are key tools for patient drug safety 9,10.

Automated dispensing devices (ADDs) are increasingly present in healthcare organizations. The transition of the pharmaceutical profession to direct patient care, changes in healthcare systems and cost-reduction pressures have promoted the use of ADDs. The American Society of Health-System Pharmacists (ASHP) approves the use of automated drug distribution because the system frees pharmacists from labor-intensive distribution functions. Those pharmacists then begin to share responsibility for drug inventory with nurses, which improves the accuracy and timeliness of drug availability and enhances patient care 11.

A recent systematic review showed that ADDs decrease drug storage errors and increase resource management efficiency. The nursing staff spent significantly less time managing controlled drugs. The results related to other drugs are inconclusive. Similarly, there was no clear evidence that these devices reduced drug errors resulting in patient harm, increased nursing or pharmacy staff time spent on patient care or reduced hospital costs 12.

Although the literature indicates a high usage rate of this technology in tertiary hospitals within developed countries, studies specifically addressing ADDs in Brazilian hospitals remain scarce.

This study was conducted to assess the economic and patient-safety impact of the implementation of the Pyxis® automated materials and drug-dispensing system in an inpatient unit for critically and semi-critically ill patients in a tertiary hospital.

## METHODS

### Study site, period and design

The study was conducted at “Hospital Sírio-Libanês” (HSL), which is a private, tertiary hospital that has 368 beds, including 44 intensive care beds; averages 18,840 admissions per year; and is located in São Paulo, Brazil. The hospital pharmacy department consists of a central pharmacy, 23 storage units, and 5 satellite pharmacies located in the following specific hospital units: the oncology unit, the emergency room, the surgery unit, the intensive care unit, and the diagnostic and imaging center. The hospital adopted an individual medication order-distribution system. The oncology department and the main intensive care unit used the unit-dose system.

The Pyxis® ADD system was gradually introduced at HSL units in São Paulo from 2013 to 2015, primarily to promote drug-use safety by integrating drug prescription with dispensing. This new technology was also expected to reduce the time spent by the nursing and administrative staff on managing the inventory of the drug room and to improve urgent care performance. Improvements in the quality of patient billing (through the adequate invoicing of drugs and disposable medical supplies in the unit) and stock accuracy to reduce inventory adjustments were also expected.

The effect of introducing the ADD system for the storage and control of the peripheral stock located in an 11-bed unit housing semi-critically and critically ill patients was assessed. Clinical and cost outcomes were assessed with cost outcomes considering a 5-year analytical horizon starting in 2013.

The characterization of the central pharmacy regarding the products administered, the products dispensed, and the number of professionals in 2013 is outlined in Table 1.

### Intervention

The Pyxis® ADD system was pilot-deployed at an HSL unit in September 2013.

The unit in which the Pyxis® was implemented consisted of eleven cardiology beds, including seven semi-critical beds and four intensive care beds. The demographic characterizations of the unit in the periods before (January-August 2013) and after (October 2013-October 2014) the introduction of the ADD system were similar.

Drug dispensing via the HSL pharmacy was performed using a hybrid model. Regularly prescribed drugs were dispensed by the central pharmacy. Drugs and disposable materials used during the first hours of patient admission as well as those that are used because of changes in drug prescriptions were dispensed from the peripheral stock located within each care unit. Each care unit also had a cart stocked with drugs and materials for emergency care.

The patient profile and medical specialties of the care unit determined the composition of the drugs and disposable materials in this peripheral stock. The quantity of each product was based on the historical quantitative consumption provided by the hospital information system.

The products were barcoded to ensure the traceability, stock write-off, and invoicing of the drugs and materials included in the peripheral stocks.

The peripheral stock structure changed significantly with the implementation of the ADD system. Previously, products were placed in conventional cabinets in the drug room in the care unit, and only controlled drugs were stored in lockable drawers 13. The other products were stored in various places, according to the convenience of the team managing the stock. Resupply was performed once per day and was automatically generated by the hospital information system. Electronic prescription was not integrated with this stock, which together with the possibility of introducing or removing products from cabinets without a mandatory barcode reading, led to the need for many inventory adjustments and increased potential error. Administrative processes (invoicing, stock write-off for resupply and control, and key procedures in drug use safety) depended on the adherence of the nursing staff (who were directed to access the hospital information system using a password) to select the patient and perform the barcode reading of each product.

After introducing the ADD system, the peripheral stock began to be placed inside the device within the care unit, resulting in a centralized, organized, and closed stock. Resupply, which was automatically generated by the hospital information system, began to be performed twice daily and was monitored by the team of pharmacy assistants to ensure organization according to the record of each product in the dispensing system. Electronic prescriptions were integrated with the dispensing system with biometric control of stock access for all users and privileges according to professional category. From that time forward, the nursing team had access to only the drugs prescribed for each patient. Emergency drugs, such as those not reviewed by the pharmacist and not part of the patient’s profile, were removed only by a nurse who was previously authorized to execute an override order. Then, the patient and the drugs were selected based on time, and each compartment containing the drug or material opened only for the practitioner to remove the products from the dispensing system after all of these steps were completed. After closing each compartment, the system invoiced the patient and adjusted the inventory. No change was introduced regarding the control of product expiration dates, which was performed monthly by the pharmacy team.

### Data analysis

The following outcomes were considered in the analysis: number of ADEs, audit adjustments to invoicing of materials and drugs in patient bills, performance in meeting urgent requests, need for central pharmacy services and product returns from the unit to the central pharmacy.

According to the World Health Organization, an ADE is a patient injury resulting from a medication, either because of a pharmacological reaction to a normal dose or because of a preventable adverse reaction to a drug resulting from an error 14. Data concerning ADEs were collected from the HSL information system, which stored the details of each event notification, including the date, place, type of occurrence, drug involved, phase of the process, classification, and degree and type of resulting harm. Events that occurred in the unit during the study period were analyzed. Events that occurred during the drug prescription and transcription phases were disregarded. The events were categorized by phase of the process (dispensing or administration) and harm classification. Harm to patients was classified in accordance with the World Health Organization International Classification for Patient Safety. Mild harm was characterized as an event that results in mild symptoms, loss of function, and minimal or intermediate damage, albeit short-lived, without need for major intervention or requiring minimal intervention (observation, investigation, review, or minimal treatment). Moderate harm was characterized as an event resulting in symptoms that required intervention (additional surgical procedure or therapeutic treatment), prolonged hospital stay, permanent long-term damage or loss of function. Severe harm was defined as a symptomatic event requiring life-saving intervention or a major surgical/medical intervention, shortened life expectancy, or major permanent or long-term harm or loss of function.

The time spent by the healthcare professionals involved in the drug and material management process of the care unit was assessed directly by measuring the work time of each activity and healthcare professional involved. This measurement was performed before and after implementing the ADD system. This assessment was performed by a pharmacist who was an HSL pharmacy coordinator.

The working time was then estimated as hours per day spent on each activity. The cost per hour of work for each professional category was estimated from the gross monthly salary (including the benefits paid by the hospital), considering a weekly load of 40 hours. In addition, 30% pay for vacation and a 1 month salary bonus at the end of every working year (i.e., a 13^th^ paycheck) were used to estimate annual salary because these benefits are paid by the hospital per Brazilian legislation. The working time per day was multiplied by the number of days in a year to estimate the annual economic impact. The total annual time (in hours) was multiplied by the salary per hour of work resulting in the annual costs for personnel by professional category. Personnel costs during the period before the implementation of the ADD system were then compared with personnel costs during the period after the implementation of the ADD system.

The periods pre- and post-introduction of the automated drug-dispensing system were considered January to August 2013 and October 2013 to October 2014, respectively, for all outcomes.

Categorical data are described and expressed as absolute (n) and relative (%) frequencies. Continuous variables are described as the mean and standard deviation. Unpaired Student’s t-test was used to compare the means of continuous variables with normal distributions. Pearson’s chi-squared test was used to compare ratios. The significance level was set at 0.05 for all tests. The data were tabulated and analyzed using Microsoft Excel 2010 and SPSS for Windows, version 22.

Direct medical costs, including the investment costs for implementing the Pyxis® ADD system and human resource costs, were considered in the cost analysis. All costs were expressed as Brazilian Reals (R$) and converted to US Dollars (USD) considering the official exchange rate in December 2013 (1 BRL=0.43 USD). To estimate the annual cost of human resources for future years (2014-2017), the estimated annual savings resulting from averted costs for human resource salaries for 2013 were then adjusted for inflation considering the Brazilian annual national consumer price index 15 according to the salary-adjustment procedures adopted by the hospital. The following annual cumulative national consumer price indices were used: 5.56% for 2013, 6.23% for 2014, 11.28% for 2015, and 6.58% for 2016.

The rate of medical equipment depreciation was set at 10% per year, assuming a useful life of 10 years, according to current Brazilian law 16.

The study was reviewed by the Institutional Review Board, which waived the need for approval.

## RESULTS

### Number of events

During the study period, a total of 37 ADEs were reported in the unit. Most (n=31, 83.8%) adverse events occurred during the drug-dispensing phase, whereas the other events (n=6, 16.2%) occurred during the drug administration phase.

Although a decrease in the mean number of events reported was observed between the ADD system pre- (2.25±2.19 events/month) and post-implementation (1.46±1.39 events/month) periods, this difference was not significant (*p*=0.32). The same trend was also observed when assessing the events that occurred during drug dispensing (1.88 *versus* 1.23, *p*=0.34) and administration (0.38 *versus* 0.23, *p*=0.65) separately. It is worth noting that the numbers are very small, thus limiting the power of the comparative analysis of means between the two periods.

### Audit adjustments to hospital bills

The number of audit adjustments to the hospital bill of each hospitalized patient was analyzed regarding the inclusion or exclusion of materials and drugs administered.

We observed a significant decrease in adjustments to drug inclusions and exclusions and material inclusions. A decrease in material exclusions also occurred, although this change was not significant (Table 2).

### Product requests and returns to the central pharmacy

A significant decrease (by 71%) in urgent requests was observed after implementing the ADD system when assessing the number of requests and the need for central pharmacy services during both periods (Table 3). Similarly, the number of products returned to the central pharmacy decreased significantly (by 30%) during the ADD system post-implementation period. The observed 15% decrease in routine requests was not significant.

### Healthcare professional time

The implementation of the ADD system resulted in a change in the distribution of the time and type of healthcare professionals involved in drug management activities (Table 4). The time spent on activities performed by nurses and administrative assistants decreased, whereas the time spent on activities performed by pharmacy assistants increased, resulting in a total reduction of 6.5 work hours per day (Table 4).

**Figure 1:**
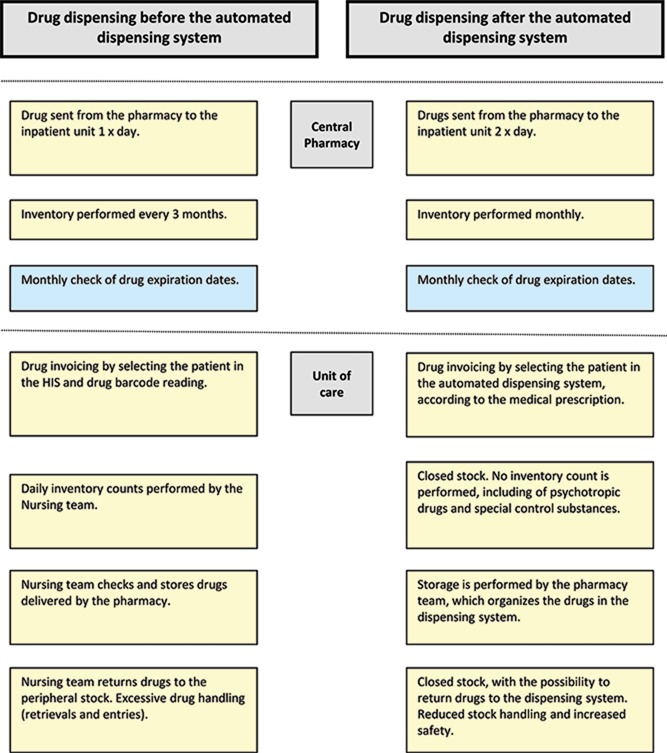
Drug dispensing before and after implementing the automated dispensing system. Hospital Sírio-Libanês, São Paulo. Brazil. 2013-2014.

Nursing time was devoted primarily to drug inventory activities, including counting controlled drugs and performing stock write-offs, which significantly decreased after the implementation of the ADD system. Administrative assistant time, which also decreased, was primarily devoted to counting drugs and materials and resupplying items (Figure 1).

Stock inventory and item resupply activities, which were performed by the stock room supervisor and administrative assistants, respectively, began to be performed by another professional, i.e., the pharmacy assistant.

### Costs

The total cost of the Pyxis® ADD system included the cost of the device (R$ 198,065.88; USD 85,153) and costs associated with cabinet-making and remodeling services (R$ 8,000.00; USD 3,439.40). Information technology (IT) costs were disregarded because the server was included in the cost of the device and the user interface software between the central pharmacy computer system and the ADD system was provided by the IT service of the HSL.

The 2013 average mid-career salary, considering the gross monthly salary for a 40-hour weekly workload, was used in the analysis of healthcare personnel costs as follows: full-time nurse (R$ 5,869; USD 2,523), administrative/hospitality assistant (R$ 1,598; USD 687), materials supervisor (R$ 3,526; USD 1,516), and pharmacy assistant (R$ 1,710; USD 735).

Thus, the reduction in personnel costs totaled R$ 33,598 (USD 14,444) per year during the first year after introducing the ADD system (Table 5).

For the 5-year period between 2013 and 2017, considering the salary adjustment based on the Broad National Consumer Price Index between 2013 and 2016 and assuming a constant inflation rate of 10% starting in 2016, the reduction in personnel costs alone totaled R$ 35,480 (USD 15,254) in 2014, R$ 37,690 (USD 16,204) in 2015, R$ 41,942 (USD 18,032) in 2016, and R$ 44,702 (USD 19,218) in 2017. Thus, the initial investment would be paid off in 5 years, considering only personnel savings.

## DISCUSSION

This investigation is the first study to assess the impact and costs resulting from implementing automated dispensing systems in Latin America. Implementing this technology resulted in reduced human resource costs, reduced audit adjustments regarding drug inclusions and exclusions and materials inclusions, and a lower number of urgent requests and product returns to the central pharmacy.

Our results corroborate evidence from developed countries. Overall human resource time and costs decreased because of a reduction in nursing time and activity assignment, whereas the work time of pharmacy assistants increased. A study assessing the implementation of the Pyxis® system in the United States in 1995 showed a significant reduction in personnel time related to the use of the ADD system that resulted in a savings of approximately USD 1 million during a 5-year period for the institution 17. Similar findings were observed in Spain, where the authors reported significant increases in clinical activities by pharmacists despite not having identified significant reductions in medication-related activities 18. This result might be related to the type of work attributed to pharmacists and nurses; however, it also reinforces the finding that the ADD system provides pharmacists with more time for other types of clinical work.

Other studies have assessed the economic effect of ADDs using various methods 19-24. Although those authors showed a reduction in human resource personnel time and costs, in the United States, Klein et al. 24 concluded that this reduction was insufficient because the costs of the bulk drugs purchased to supply the local ADD system were higher than those for the same drugs in conventional packaging. However, the drugs in Brazil are included in the ADD system in unit-dose packaging, which is the same as in manual systems.

Financial impact studies of ADDs in Spain 21-23 have indicated a reduction in workload and personnel costs. Poveda et al. 23 conducted a cost-benefit analysis for the implementation of 11 ADDs in clinical and surgical intensive and emergency care units of a university hospital center consisting of two hospital units in Albacete, Spain. These authors found a positive cost-benefit ratio (1.95) that favored the implemented technology. Another publication from the same study that considered a longer follow-up time reported an estimated €32,390 in annual personnel savings, which resulted in an even higher cost-benefit ratio (2.19); in that case, the initial investment was paid off in an estimated 44 months 21.

This result is similar to the findings of our study, which estimated that the initial investment would be paid off in 5 years or 60 months, considering the reduction in personnel costs alone.

Although the aforementioned studies consistently indicated the positive economic impact of implementing ADD systems, a systematic review conducted by the Canadian Agency for Drugs and Technologies in Health (CADTH) in 2009 to assess the impact of new technologies for drug dispensing and administration regarding their effectiveness, cost-effectiveness and budget impact indicated a lack of robust and high-quality evidence related to ADD systems 25,26.

Since that time, other studies have been performed using the before-and-after method and were therefore subject to various biases. A study assessing the economic effect of implementing an ADD system in a 12-bed resuscitation/intensive care unit in a French university hospital using the cash flow method showed reductions in personnel time costs and drug-storage costs. This cost reduction resulted in an estimated 5-year savings of €71,586 19,20.

A more complete and recent analysis of the financial effect of ADD use in a surgical intensive care unit of a French university hospital also showed a decrease in drug-related nursing time and an increase in time dedicated by pharmacy technicians to the local stock. This study also considered the decrease in the stock of expired drugs and their respective costs. The annual cost of the drug stock decreased by €44,298, and the cost of expired drugs decreased by €14,772. Using the cash flow method, the study showed that the overall cash flow was €148,229, and the current net value of the project 5 years after the initial investment was positive by €510,404. The authors concluded that the implementation of ADD systems results in a high return on the initial investment 19.

The impact of the ADD system on the number and value of drugs in stock or on expired drugs could not be assessed in our study. It is worth noting that most institutions use the model of fully meeting drug needs using the stock of the ADD system. In the “hybrid” model implemented at the HSL described above, the peripheral stocks available in the care units had minimal product types and enough quantity to minimize financial losses and care failures. The implementation of the ADD enabled the unit to increase the number and availability of certain types of products, thereby increasing process agility.

Packaging, dispensing, and barcoding prescribed items using an ADD system for prescription items can decrease the occurrence of ADEs 10. Although a decrease in the number of ADEs was observed in this study, this decrease was non-significant. Considering that the number of events that occurred during the study period was small, this finding might result from the low power of the study, which failed to show an effect on ADEs. However, recently published evidence indicates controversy regarding the effectiveness of ADD systems to reduce ADEs related to drug administration. Only one of 3 systematic reviews published recently showed that ADD systems effectively reduce the number of ADEs, albeit modestly (RR=0.72, therefore, 28% effectiveness) 27.

The other studies reported no evidence of decreased ADEs from using this technology. However, all reviews emphasized the small number of studies and the high risk of bias in the studies reviewed 28.

Thus, similar to most studies on this subject, our study has several limitations that should be reported. First, the study was performed retrospectively and using secondary data available in the hospital. Thus, several outcomes of interest, including stock control and expired drugs, could not be assessed, as mentioned previously. Furthermore, costs related to the necessary planning, training and information system adaptations in the stage of preparation for implementing the automated drug-dispensing system were not included in the cost analysis.

Despite these limitations, the preliminary results from this study support the decision to gradually implement the automated Pyxis® drug-dispensing system in another 16 HSL units starting in 2013.

Previous studies have indicated the widespread use of ADD systems in several countries. In the United States, a nationwide survey conducted by the American Society of Health-Systems Pharmacists in 2011 found that 89% of the 1,401 hospitals assessed used ADD systems 29. A Spanish study from 2013 on the adoption of safe drug practices included 36 hospitals and showed that 60.9% of the hospitals used ADD systems coupled with electronic prescription systems 30.

In Brazil, reports indicate that this technology has been gradually incorporated into hospital departments. However, little evidence on this subject is available in the literature. Although this study was performed in a tertiary hospital located in São Paulo (and therefore precludes direct extrapolations of the results to other hospitals, particularly Brazilian private hospitals), we believe that our results are relevant, especially in the Brazilian context, considering the lack of studies on the subject.

The results from this study contribute to the body of available evidence showing the positive effect of ADD systems in reducing healthcare personnel time and costs and other outcomes of interest related to healthcare services. Studies assessing the cost and effect of implementing technologies related to pharmaceutical care in hospital departments remain scarce in Brazil and should be encouraged, considering the importance of their results to the decision-making process. This analysis, in the context of a Brazilian tertiary private hospital, is useful and may help Brazilian healthcare decision-makers and managers regarding the use of this technology.

## AUTHOR CONTRIBUTIONS

de-Carvalho D and Alvim-Borges JL conceived and designed the study. Toscano CM designed the study methods as well as analyzed and interpreted the data. All authors were responsible for writing the manuscript and have approved the final version.

## Figures and Tables

**Table 1:** Characterization of the Central Pharmacy of Hospital Sírio-Libanês regarding the products administered and dispensed as well as the number of staff. 2013. São Paulo, Brazil.

Characteristic	Monthly mean (number)
**Products administered**	**1,994,973**
**Products dispensed**	** 270,421**

Professionals	Number of professionals
Pharmaceutical manager	1
Pharmacy coordinators	4
Materials supervisors	5
Pharmacists	35
Pharmacy technicians	11
Pharmacy assistants	102
Messengers	12
Assistants and administrative assistants	38
Trainees	4
Young apprentices	1
Total	213

**Table 2:** Audit adjustments to material and medication invoicing in the Advanced Heart Failure Unit of Hospital Sírio-Libanês before and after implementing the automated medication dispensing system. 2013-2014. São Paulo, Brazil.

Type of product	Procedure requested by the audit	Conventional dispensing period		Automated dispensing period		Variation (%)	*p*-value
		Mean	Standard deviation	Mean	Standard deviation		
Drugs	Inclusion (mean/month)	1,215.75	815.49	555.08	210.49	-54.30	0.01
	Exclusion (mean/month)	1,635.13	451.19	1,036.92	297.21	-36.60	<0.01
Materials	Inclusion (mean/month)	1,061.25	668.20	461.54	151.72	-56.50	<0.01
	Exclusion (mean/month)	3,008.75	1,425.01	2,255.15	786.20	-25.10	0.13

**Table 3:** Urgent and routine requests as well as pharmaceutical product returns to the central pharmacy at Hospital Sírio-Libanês before and after implementing the automated medication dispensing system. 2013-2014. São Paulo, Brazil.

	Conventional dispensing period		Automated dispensing period		% Reduction	*p*-value
	Mean; SD (R$)	Mean; SD (USD)	Mean; SD (R$)	Mean; SD (USD)		
Total requests/month						
Routine	1,510 (332.60)	649.18 (142.99)	1,279 (385.10)	549.87 (165.53)	-15.3	0.17
Urgent	1,519 (220.90)	279.1 (94.97)	431 (88.90)	185.30 (38.22)	-71.6	<0.001
Total returns/month	869 (90.50)	373.60 (38.91)	603 (136.30)	259.11 (58.60)	-30.60	<0.001

SD = standard deviation.

**Table 4:** Activity and time associated with healthcare professional involvement in drug and material management in the Advanced Assistance Unit of the Hospital Sírio-Libanês before and after implementing the automated medication dispensing system, according to the type of professional. 2013-2014. São Paulo, Brazil.

Type of healthcare professional and activities	Conventional dispensing period	Automated dispensing period	Variation	
	Hours/day	Hours/day	Hours/day	%
Nurse	3.75	0	-3.75	-100
Daily inventory count – controlled substances	2.25	0	-2.25	-100
Adjustments/ corrections of the inventory counts	1.5	0	-1.5	-100
Material supervisor	0.07	0	-0.07	-100
Stock inventory	0.07	0	-0.07	-100
Administrative assistant	5	2	-3	-60
Daily inventory count – drugs	2	0	-2	-100
Daily inventory count – materials	2	0	-2	-100
Item resupply	1	2	1	100
Pharmacy assistant	0	4	4	New
Item resupply	0	1	1	New
Stock inventory	0	3	3	New

**Table 5:** Human resource costs of the Advanced Assistance Unit of the Hospital Sírio-Libanês before and after implementing the automated medication dispensing system, according to the type of healthcare professional. 2013-2014. São Paulo, Brazil.

Type of healthcare professional	Conventional dispensing period				Automated dispensing period				Variation/ year	
	Cost/day (R$)	Cost/day (USD)	Cost/year (R$)	Cost/year (USD)	Cost/day (R$)	Cost/day (USD)	Cost/year (R$)	Cost/year (USD)	R$	USD
Nurse	101.64	43.70	36,589.55	15.730.67	0.00	0.00	0.00	0.00	-36,589.55	-15,730.67
Materials supervisor	1.14	0.49	410.34	176.41	0.00	0.00	0.00	0.00	-410.34	-176.41
Administrative assistant	36.90	15.86	13,283.38	5,710.82	14.76	6.35	5,313.35	2,284.33	-7,970.03	-3,426.49
Pharmacy assistant	0.00	0.00	0.00	0.00	31.59	13.58	11,371.50	4,888.87	11,371.50	4,888.87
TOTAL	139.68	60.05	50,283.26	21,617.91	46.35	19.93	16,684.85	7,173.19	-33,598.41	-14,444.72
